# Ion Transporters and Osmoregulation in the Kidney of Teleost Fishes as a Function of Salinity

**DOI:** 10.3389/fphys.2021.664588

**Published:** 2021-04-20

**Authors:** Marius Takvam, Chris M. Wood, Harald Kryvi, Tom O. Nilsen

**Affiliations:** ^1^Department of Biological Sciences, University of Bergen, Bergen, Norway; ^2^NORCE, Norwegian Research Centre, NORCE Environment, Bergen, Norway; ^3^Department of Zoology, University of British Columbia, Vancouver, BC, Canada

**Keywords:** physiology, renal function, osmoregulation, euryhaline teleosts, salinity, ion transporters

## Abstract

Euryhaline teleosts exhibit major changes in renal function as they move between freshwater (FW) and seawater (SW) environments, thus tolerating large fluctuations in salinity. In FW, the kidney excretes large volumes of water through high glomerular filtration rates (GFR) and low tubular reabsorption rates, while actively reabsorbing most ions at high rates. The excreted product has a high urine flow rate (UFR) with a dilute composition. In SW, GFR is greatly reduced, and the tubules reabsorb as much water as possible, while actively secreting divalent ions. The excreted product has a low UFR, and is almost isosmotic to the blood plasma, with Mg^2+^, SO_4_^2–^, and Cl^–^ as the major ionic components. Early studies at the organismal level have described these basic patterns, while in the last two decades, studies of regulation at the cell and molecular level have been implemented, though only in a few euryhaline groups (salmonids, eels, tilapias, and fugus). There have been few studies combining the two approaches. The aim of the review is to integrate known aspects of renal physiology (reabsorption and secretion) with more recent advances in molecular water and solute physiology (gene and protein function of transporters). The renal transporters addressed include the subunits of the Na^+^, K^+^- ATPase (NKA) enzyme, monovalent ion transporters for Na^+^, Cl^–^, and K^+^ (NKCC1, NKCC2, CLC-K, NCC, ROMK2), water transport pathways [aquaporins (AQP), claudins (CLDN)], and divalent ion transporters for SO_4_^2–^, Mg^2+^, and Ca^2+^ (SLC26A6, SLC26A1, SLC13A1, SLC41A1, CNNM2, CNNM3, NCX1, NCX2, PMCA). For each transport category, we address the current understanding at the molecular level, try to synthesize it with classical knowledge of overall renal function, and highlight knowledge gaps. Future research on the kidney of euryhaline fishes should focus on integrating changes in kidney reabsorption and secretion of ions with changes in transporter function at the cellular and molecular level (gene and protein verification) in different regions of the nephrons. An increased focus on the kidney individually and its functional integration with the other osmoregulatory organs (gills, skin and intestine) in maintaining overall homeostasis will have applied relevance for aquaculture.

## Introduction

Several teleost fish species have developed strategies to maintain fluid and electrolyte homeostasis in a wide range of salinities, involving integrated ion and water transport activities of the gills, kidney and intestine (Evans et al., 2005; Marshall and Grosell, 2005; Evans, 2010; Grosell, 2010). By definition, stenohaline fish are able to cope only within a narrow salinity range in either freshwater (FW) or seawater (SW) (Gonzalez, 2012). Approximately 3–5% of teleosts are euryhaline with the ability to acclimate to strongly hypotonic and hypertonic salinities (Evans, 1984). Anatomical and physiological knowledge of the cooperative osmoregulatory functions of gills, kidney and intestine is abundant (Marshall and Grosell, 2005; Evans, 2008, 2010; Grosell, 2010; Hwang et al., 2011; Whittamore, 2012). Hence, major reviews have focused on renal physiology in FW and SW acclimated fishes (Hickman and Trump, 1969; Wood and Patrick, 1994; Wood, 1995; Renfro, 1999; Beyenbach, 2004; Dantzler, 2016; McCormick et al., 2013a), while renal function has been addressed to a larger extent in mammals as dysfunctional regulation of water and ions is linked to many pathological conditions (Markovich, 2001; Dawson et al., 2003; Markovich and Aronson, 2007; Arjona et al., 2019; Chen et al., 2020). The purpose of the current review is not to reiterate fine details already covered in the above-mentioned reviews, but rather to integrate known aspects of renal physiology with emerging genetic and molecular information on the transporters in the kidney of euryhaline teleosts. Euryhaline teleosts are especially appealing because: (1) it is important to understand coping mechanisms of fish in the face of global climate change, because rising sea levels due to the polar ice melting are associated with decreased ocean salinity in pelagic zones. Conversely, increased salinization of coastal areas is occurring as a consequence of extreme climate events (floods, tsunamis, hurricanes, etc.) where seawater often invades freshwater environments (Kültz, 2015), and, thus, may negatively affect stenohaline fish that normally regulate within a narrow salinity range, (2) whole genome duplication (WGD) has occurred four times during evolution, where WGD3 (teleost-specific) and WGD4 (salmonid-specific) have generated new genomic material in teleosts that may provide increased phenotypic diversity (Kondrashov et al., 2002; Kondrashov, 2012; Macqueen and Johnston, 2014; Houston and Macqueen, 2019), (3) recent studies have revealed some of the molecular basis of the water and ion transport processes identified in previous physiological characterization of the euryhaline teleost kidney, and (4) among several phyla such as salmonids, eels and cichlids, which are critically important for global fisheries and aquaculture, there are many species with declining wild populations. Our overall aim is to highlight recent advances in molecular transport pathways and their role in the renal function of euryhaline teleosts.

## Genome Plasticity of Euryhaline Teleosts and Anatomical Characteristics of the Kidney

Salinity tolerance is a careful balance between bioenergetic costs and trade-offs that ultimately may modulate adaptive plasticity and evolutionary change in kidney function (Watt, 1985; Schulte, 2001; Dalziel et al., 2009). Many novel paralogous genes associated with water and ion regulation have been discovered in teleosts compared to the mammalian models, presumably enabling teleosts to inhabit and thrive in different aquatic habitats (Cutler and Cramb, 2001; Kondrashov et al., 2002; Takei et al., 2014; Warren et al., 2014). WGD events have generated thousands of duplicate genes, argued to be one of the major drivers for shaping the vertebrate genome during evolution (Sato and Nishida, 2010). Euryhaline teleosts such as salmonids exhibit a high paralog retention (25–75%), often exhibiting transporter paralogs not found in other teleosts, leading to more extensive genomic plasticity (Bailey et al., 1978; Houston and Macqueen, 2019). The plasticity originating from the teleosts specific WGD3 events approximately 320-350 million years ago (mya) and salmoniformes WGD4 events 50-80 mya may have enhanced the capacities for euryhaline species in these phyla to deal with salinity fluctuations and to acclimate to FW and SW environments (Houston and Macqueen, 2019).

**FIGURE 1:**
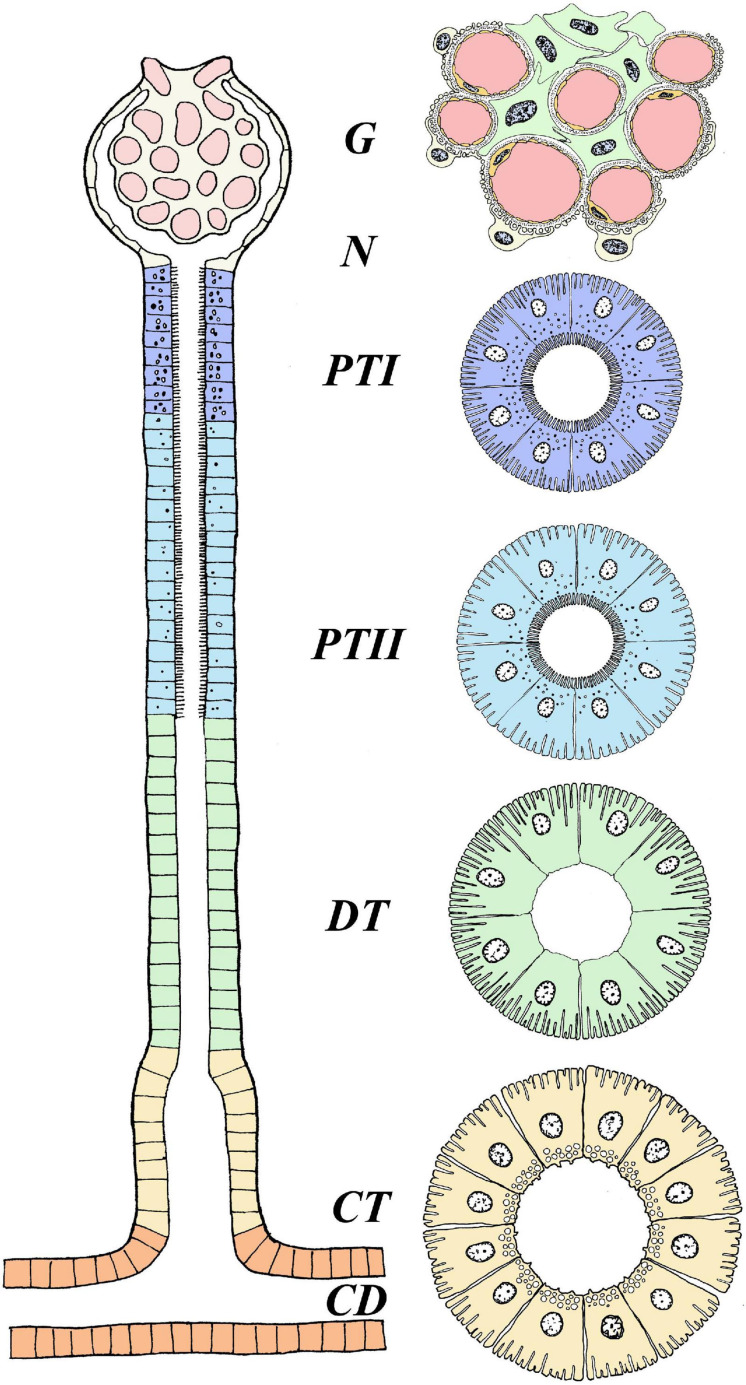
Anatomical overview of the nephron in euryhaline teleosts. The figure represents the general anatomy of the nephron in euryhaline species with the following segments: Glomerulus (G), Proximal Tubule I (PTI), Proximal Tubule II (PTII), Distal tubule (DT) and Collecting tubule (CT). Hence, no cross section overview is given for neck segment (N) and collecting duct (CD). Note that the intermediate segment, only found in FW stenohaline teleosts, is not illustrated. The anatomical differences with well-defined vacuoles and microvilli in the proximal tubule (more distinct in PTI), basolateral membrane infoldings in all tubule segments (more distinct in DT) and apical located membrane granules in CT. Model are based on Hickman and Trump (1969). Further note that this illustrates the main anatomical features of the nephron, and that differences may exists between species. For a more comprehensive detailed anatomical view see Hickman and Trump (1969) and Dantzler (2016).

In their seminal review, Hickman and Trump (1969) provided a detailed overview of the evolution and anatomy of the teleost kidney. Both microscopic observations and studies on isolated tubules divide the nephron of euryhaline fishes into sections: glomerulus (excluding aglomular fish), proximal tubule I and II (tubule II is longer, and marine teleosts may have a third proximal tubule), intermediate segment (only present in freshwater teleosts), the distal tubule (sometimes missing in marine teleosts), and the collecting tubule and collecting duct (Hickman and Trump, 1969; Figure 1). The anatomical and regulatory properties of these segments may differ depending on the salinity the fish is acclimated to, or if the animal is transitioning between salinities.

In euryhaline species, glomeruli and tubular segments are tangled and in close connection with hematopoietic tissue, especially in the anterior part of the kidney, while urine producing nephrons are more numerous toward the posterior part (Anderson and Loewen, 1975; Resende et al., 2010). Afferent arterioles enter into a network of capillaries at the vascular pole of the glomerulus. In these capillaries, the arterial blood pressure filters the plasma through small pores into Bowman‘s capsule, consisting of visceral and parietal layers (Brown et al., 1983). The internal or visceral layer of the glomerulus has modified epithelial cells, called podocytes (“foot cells”) with intermingling projections (pedicels) that wrap around the glomerular capillaries, with narrow slits between them. These, together with the intercellular channels (“fenestrae”) of the vascular endothelium, and a trilaminar basement membrane create a filtration barrier (“sieve”) that is small enough to allow the passage of both essential and unwanted solutes from the blood, yet not large enough for proteins or red blood cells (RBC) to permeate (Elger et al., 1984). Plasma filtered into Bowman’s capsule is the primary urine, almost identical in composition to the blood plasma from which it was formed, apart from the absence of proteins. The primary urine passes the short neck segment and thereafter, is sequentially modified by reabsorption and secretion processes as it passes through the proximal and distal tubules before entering the collecting tubule and duct (Charmi et al., 2010). Ultimately, the urine enters the paired mesonephric duct which merge together, forming the urinary bladder where the urine is stored before it is periodically discharged through the urinary papilla (Curtis and Wood, 1991; Demarest and Machen, 1984). All parts of the nephron tubules and urinary bladder exhibit transport processes that contribute to the final volume and composition of the urine.

## Renal Handling of Ions and Water in FW and SW Environments

**FIGURE 2:**
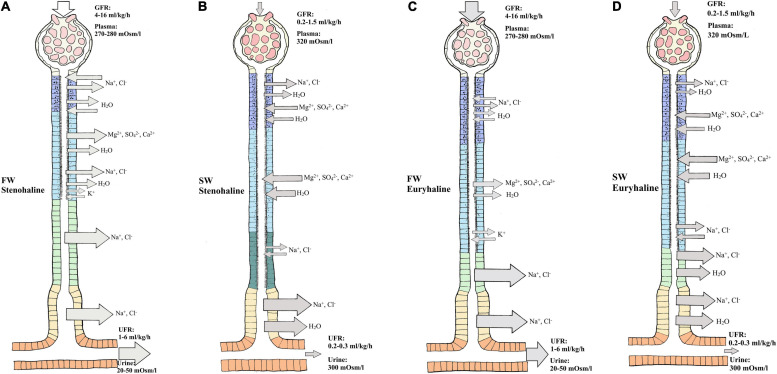
Overview of water and ion movement in freshwater teleost nephron (FW) and seawater teleost nephron (SW). FW **(A,C):** In a FW fish kidney, most ions are reabsorbed, and water follows by diffusion. Therefore, impermeable features of the distal and collecting duct can reabsorb Na^+^ and Cl^–^ with minimal accompanying osmotic movement. The glomerulus filters (GFR) roughly 4–16 milliliter/kilogram/hours (ml/kg/h) of plasma (270–280 mOsm/L) while producing (UFR) roughly 1-6 ml/kg/h of dilute urine (20–50 mOsm/L). SW **(B,D):** In a SW fish kidney, most divalent ions are secreted and water follows by osmosis. To counter water loss and dehydration in SW fish, distal and collecting tubule must be more permeable enabling effective Na^+^ and Cl^–^ reabsorption while water can follow by osmosis. The glomerulus filters (GFR) roughly 0.2–1.5 ml/kg/h of plasma (320 mOsm/L) while producing (UFR) roughly 0.2–0.3 ml/kg/h of concentrated urine (300 mOsm/L) rich in SO_4_^2–^ and Mg^2+^. Different sections of the nephron are displayed as follows: Glomerulus (G), Neck segment (NS), Proximal tubule I (PTI), Proximal tubule II (PTII), Distal tubule (DT), Colleting tubule (CT) and collecting duct (CD). Keep in mind that the nephron in euryhaline teleost in FW and SW environments usually has a distal segment (model C and D) commonly found in stenohaline FW teleosts (Model A) while stenohaline SW teleosts (model B) possess a third proximal segment. Transport in the collecting duct are not indicated here but are known to have similar roles as in the collecting tubule. Models modified from the paper of Hickman and Trump (1969) and Marshall and Grosell (2005).

In FW, ions are scarce and Osmolarity is very low, so stenohaline (FW) and euryhaline (when in FW) fishes experience a continuous osmotic influx of water and diffusive loss of major ions through the gills and skin. The kidney counters this by filtering large amounts of blood in the glomeruli, thereby maintaining high glomerular filtration rates (GFR) of approximately 4–16 ml//kg/h and urine flow rates (UFR) of 1–6 ml/kg/h, ensuring excretion of large volumes of dilute urine (20–50 mOsm/L) (Hickman and Trump, 1969; Beyenbach, 1995; (Figures 2A,C). The urine of FW teleosts typically contains 5–20 mM of NaCl with other ions being at most a few millimolar (mM), and often in the sub-millimolar range (Hickman and Trump, 1969). Production of dilute urine is possible due to the impermeable features of the distal tubule and downstream regions (collecting tubule/duct), including the bladder, enabling the reabsorption of precious ions (mainly Na^+^ and Cl^–^) while limiting the accompanying osmotic reabsorption of water. In proximal tubule I, organic acids are secreted, while compounds like glucose, other macromolecules and NaCl are reabsorbed, accompanied by some water (Hickman and Trump, 1969; Marshall and Grosell, 2005; Figures 2A,C). Although minor secretion of Mg^2+^ and SO_4_^2–^ occurs (Nishimura et al., 1983), most divalent ions such as Mg^2+^, SO_4_^2–^, and Ca^2+^ are reabsorbed in proximal tubule II of the nephron. Na^+^, Cl^–^, K^+^, and HCO_3_^–^ are also reabsorbed in proximal tubule II, together with some water (Cliff and Beyenbach, 1992; Dantzler, 2003). Although the primary function of the FW kidney is solute reabsorption and water excretion, most measurements to date have been on fasted fish, and evidence suggests that dietary ion intake can cause shifts from reabsorption to secretion of both monovalent and divalent ions (Oikari and Rankin, 1985; Curtis and Wood, 1991; Cliff and Beyenbach, 1992; Bucking and Wood, 2007; Bucking et al., 2010).

In SW, ions are abundant and water is lost by osmosis through the gills and skin. Fish replace water loss by drinking, but thereby incur additional Na^+^ and Cl^–^ loading which accompanies this enteric water absorption (Marshall and Grosell, 2005). In general, both urine volume and composition differ between SW and FW species. In SW, water conservation and excretion of excess divalent ions (e.g., Mg^2+^, Ca^2+^, SO_4_^2–^) by the kidney are vital. Therefore, the SW kidney typically exhibits low GFR (0.5–2.0 ml/h/kg) reflecting greatly reduced numbers of functioning glomeruli, or sometimes without glomeruli as in aglomular fish (Schmidt-Nielsen and Renfro, 1975; Brown et al., 1978), and low UFR (0.2–0.3 ml/h/kg) compared to FW fish, producing a urine which is approximately isotonic (300–410 mOsm/L) to the blood plasma. The major urinary cations are Mg^2+^, Na^+^, and Ca^2+^, while the major anions are SO_4_^2–^ and Cl^–^ (Hickman, 1968b; Hickman and Trump, 1969; Renfro, 1999; Beyenbach, 2004; Figures 2B,D). The distal tubule is usually reduced and reabsorption of Na^+^ and Cl^–^ occurs in the late proximal tubule and urinary bladder. Reabsorption of Na^+^ and Cl^–^ occurs in proximal tubule I where some water follows by osmosis (Hickman and Trump, 1969). Excretion of Mg^2+^, Ca^2+^ and SO_4_^2–^ is believed to be primarily by secretion in proximal tubule II, but there is some evidence that this also takes place in proximal tubule I (Hickman and Trump, 1969; Beyenbach, 1995; Figures 2B,D). Hence, the urine is made isosmotic largely due to reabsorption of NaCl, which is accompanied by water in the distal and collecting tubule (Dantzler, 2003; Figures 2B,D). Further reabsorption of Na^+^ and Cl^–^ appears to occur in the urinary bladder, leaving high concentrations of divalent ions in the urine (Beyenbach and Kirschner, 1975).

The preceding overview summarizes our general understanding of how the kidney functions at a macro-level in FW and SW teleosts. For additional details, the reader is referred to Smith (1930); Hickman and Trump (1969), Beyenbach and Kirschner (1975); Brown et al. (1978), Beyenbach (1986); Talbot et al. (1989), Curtis and Wood (1991); Wood and Patrick (1994), Wood (1995); Nishimura and Fan (2003), Dantzler (2003); Beyenbach (2004), Marshall and Grosell (2005), and Dantzler (2016).

## Na^+^/K^+^ - Atpase - Essential for Fluid and Electrolyte Homeostasis

Na^+^/K^+^- ATPase (NKA) maintains high and low intracellular K^+^ and Na^+^ concentrations, respectively, and is therefore crucial in the regulation of the transmembrane potential used for cellular homeostasis and secondary transport of many compounds (Skou and Esmann, 1992). Analogous to its osmoregulatory role in the gills (Evans et al., 1999; Evans, 2008; Hiroi and McCormick, 2012) and intestine (Sundell et al., 2003; Sundell and Sundh, 2012; Sundh et al., 2014), the NKA pump powers the electrochemical gradients required for reabsorption and secretion in the renal tubules (Nishimura and Fan, 2003), and indirectly enables reciprocal switchovers of ion and water transport during migrations between FW and SW (Marshall and Grosell, 2005; Edwards and Marshall, 2012).

In the kidney, the NKA plays a primary role in driving the reabsorption of Na^+^, water (indirectly) and other solutes (Hiroi et al., 2005; Tang et al., 2010; Teranishi and Kaneko, 2010; Kato et al., 2011; Hiroi and McCormick, 2012; McCormick et al., 2013a). Renal NKA activity is often responsive to changes in environmental salinity, though patterns are inconsistent amongst species. NKA activity increases in some species (European seabass (*Dicentrarchus labrax)*, wedge sole (*Dicologoglossa cuneata*), milkfish (*Chanos chanos*) upon FW to SW transfer, suggesting a heightened requirement for tubular ion transport, probably reflecting the need for secretion of divalent ions (Venturini et al., 1992; Herrera et al., 2009; Tang et al., 2010). Conversely, increased renal NKA activity upon transfer from SW to FW is also observed in striped bass (*Morone saxatilis*), spotted green pufferfish (*Tetradon nigroviridis*) and Japanese eel (*Anguilla japonica*), presumably reflecting the greater requirement of roughly 95% reabsorption of NaCl from the high volume of primary urine (Madsen et al., 1994; Lin et al., 2004; Nebel et al., 2005; Tang et al., 2012). NKA activity remaining stable in response changing salinity has also been observed in Atlantic salmon (*Salmo salar*), sea bream (*Sparus auratus*) and Senegalese sole (*Solea senegalensis*) (McCormick et al., 1989; Sangiao-Alvarellos et al., 2005; Arjona et al., 2007), perhaps reflecting more fine-tuned regulatory mechanisms. The NKA is composed of two essential subunits, the catalytic alpha (α) and the structurally important beta subunit (β), both of which are essential for modulating the transport properties of the enzyme (Kaplan, 2002). In addition, a third gamma (γ) subunit, often referred to as FXYD protein, appears to modify the kinetic properties of Na^+^ and K^+^ transport (Garty and Karlish, 2006; Tang et al., 2012; Hu et al., 2014; Yang et al., 2016). Adjustments of multiple components of the NKA enzyme provides increased regulatory plasticity, as indicated by additional paralogs detected in euryhaline teleosts (Blanco and Mercer, 1998; Richards et al., 2003; Nilsen et al., 2007; McCormick et al., 2013b). In tilapia (*Oreochromis sp.*), kidney *nka* α1 subunit mRNA expression increases in SW acclimated individuals compared with their FW counterparts (Zhu et al., 2018). In tilapia, the α-1 and α-3 subunits were the predominant isoforms expressed in SW, while the α-2 subunit was more abundant in FW fish (Yang et al., 2016). Conversely, the α-1 subunit abundance was higher in FW milkfish (*Chanos chanos*) than their SW counterparts, with no differences in α-2 and α-3 subunits (Yang et al., 2016). Expression and localization of yet undefined NKA isoforms have been found in all segments of the nephron but are more distinct in proximal II and distal segments in trout (*Oncorhynchus mykiss*), killifish (*Fundulus heteroclitus*), Japanese eel *(Anguilla japonica*) and tilapia (Katoh et al., 2008; Teranishi and Kaneko, 2010; Yang et al., 2016). Interestingly, in the Japanese eel higher NKA enzyme activity was observed in FW compared to SW, though this was not reflected by changes in *fxyd* mRNA (Tang et al., 2012). In Atlantic salmon (*Salmo salar*) renal expression of *fxyd2* and *fxyd12* was higher in FW than SW fish (Tipsmark, 2008), yet NKA enzyme activity remained unchanged (McCormick et al., 1989). Conversely, a more recent study suggested that renal FXYD12 enhanced NKA activity upon salinity challenge to maintain internal homeostasis in two euryhaline medaka species (Yang et al., 2016). Renal NKA activity was higher in FW for Indian medaka (*Oryzias dancena*) while the opposite was evident in Japanese medaka (*Oryzias latipes*). The authors linked this to an increased demand for ion reabsorption in FW and increased demand for water reabsorption and ion secretion in SW. Some of the differences in regulatory responses of subunits and enzyme activity in the kidneys of euryhaline teleosts probably reflect the life cycle history (anadromy, catadromy, amphidromy) and salinity preference. It may also stem from the use of crude tissue homogenates, obscuring regional differences. Future research should focus on segment-specific enzyme activity and expression of different subunits in relation to migratory patterns and salinity preference.

## Major Transporters of the Renal Tubules to Absorb and Secrete Monovalent and Divalent Ions

Fish live in an aquatic environment that is either hypo-osmotic or hyper-osmotic and are thus more vulnerable to changes in body fluids compared to terrestrial animals (Takei et al., 2014). As a consequence of the major ionic differences between FW and SW environments (Table 1), euryhaline species transition from high GFR/UFR to low GFR/UFR, accompanied by major alterations in tubular transport functions in which reciprocal switchovers of both water and ion transport are necessary in response to alteration in external salinites.

**TABLE 1:** Ion composition and osmolality (Osm) in freshwater (FW) and seawater (SW) and in blood plasma and urine in FW and SW teleosts.

	***Freshwater teleosts (FW)***							***Seawater teleosts (SW)***							**References**
	***Concentration (mM kg water^–1^)***							***Concentration (mM kg water^–1^)***							
	**Na^+^**	**Cl^–^**	**K^+^**	**Mg^2+^**	**SO_4_^2–^**	**Ca^2+^**	**Osm**	**Na^+^**	**Cl^–^**	**K^+^**	**Mg^2+^**	**SO_4_^2–^**	**Ca^2+^**	**Osm**	
Water	0.25	0.23	0.005	0.04	0.05	0.07	1	439	513	9.3	50	26	9.6	1050	Evans (1993)
Plasma	154	134	2.4	1.3	0.4	1.5	274	181	155	2.7	1.3	1.5	1.5	360	Miles (1971); Watanabe and Takei (2012)
Urine	28	6.4	2.1	0.42	0.008	1.1	57	17	121	1.42	133	69	19	304	Hickman and Trump (1969); Miles (1971)
GFR	4-16 ml/kilogram/hour							0.5-2 ml/kilogram/hour							Hickman and Trump (1969); Beyenbach (1995), Beyenbach (2004)
UFR	1-6 ml/kilogram/hour							0.2-0.3 ml/kilogram/hour							

*Glomerular Filtration Rate (GFR) and Urine Flow Rate (UFR) in FW and SW acclimated teleosts is also shown.*

## Monovalent Ions and the Known Transporters in the Kidney of Euryhaline Fish

Fish encounter severe ionic and osmotic gradients in both FW and SW (Table 1). While the deficits and excesses of essential monovalent ions (Na^+^, K^+^, Cl^–^) are dealt with by gills and intestine (Evans et al., 1999; Marshall, 2002; Marshall and Grosell, 2005; Grosell, 2010; Esbaugh and Cutler, 2016), the kidney also plays an important role (Beyenbach, 1986; Cliff and Beyenbach, 1992; Dantzler, 2003; Katoh et al., 2008; Kato et al., 2011). Key transport pathways are addressed below.

### Members of the Solute Carrier Family 12 (SLC12) and Kidney Specific Chloride Channels (ClC-K)

The Solute Carrier family 12 comprises part of the cation Cl^–^ transporter family, integral membrane proteins that mediate electroneutral transport of Na^+^, K^+^ and Cl^–^ across epithelial membranes (Haas, 1989). Two major NKCC isoforms occur in vertebrates, the NKCC1 (SLC12A2) and the NKCC2 (SLC12A1). NKCC2 is believed to function in an absorptive manner, and in the mammalian kidney, it is apically expressed in cells of the thick ascending limb of the loop of Henle (Haas and Forbush, 2000). NKCC1 is likely involved in transcellular Cl^–^ secretion, facilitating the entry of Cl^–^ at the basolateral membrane of proximal tubules (Dantzler, 2003). Current knowledge about kidney NKCC isoforms in euryhaline species is derived from studies of European eel (*Anguilla anguilla*, Tipsmark et al., 2002; Cutler and Cramb, 2008), killifish (Scott et al., 2004; Katoh et al., 2008), rainbow trout (Katoh et al., 2008) and mefugu (*Takifugu obscurus, Takifugu rubripes;* Kato et al., 2011). Based on these studies the teleost NKCC1 has been assigned a secretory role, while NKCC2 is thought to be responsible for reabsorption of Na^+^, K^+^ and 2Cl^–^ (Cutler and Cramb, 2002b, 2008; Lorin-Nebel et al., 2006).

Net secretion of NaCl in the proximal tubule in both FW and SW environments may be a conserved function within euryhaline teleosts (Beyenbach, 1986, 1995, 2004; Braun and Dantzler, 2011; Orlov et al., 2015). Secretion in the proximal segment is stimulated by cAMP and inhibited by the NKCC antagonist furosemide, indicating that Cl^–^ likely enters the cell on the basolateral side through NKCC1 (Beyenbach, 1995, 2004; Orlov et al., 2015). In FW-acclimated European eel, downregulation of kidney *nkcc1a* mRNA compared with SW individuals probably reflects a reduced requirement for tubular ion secretion in the proximal segment (Tipsmark et al., 2002). Studies in killifish confirm the notion that Na^+^, Cl^–^ and K^+^ are transported from extracellular fluid into the cell by basolateral NKCC (based on a general NKCC antibody), and Cl^–^, Na^+^ and K^+^ are further secreted through the apical specific Cl^–^ channels (CLC-K) and NKCC (based on a general NKCC antibody) (Katoh et al., 2008). These findings coincide well with *in vitro* studies of proximal tubules from killifish and winter flounder (*Pleuronectes americanus*, Beyenbach, 1995, 2004), suggesting a similar secretory function for proximal tubules (Cliff and Beyenbach, 1992). Conversely, the proximal segment I has also been suggested to reabsorb Na^+^ and Cl^–^ in both FW (Nishimura and Imai, 1982; Marshall and Grosell, 2005) and SW teleosts (Nishimura and Imai, 1982). Species-specific differences may account for the differences observed concerning reabsorption versus secretion of NaCl in proximal tubules or both may take place through proximal tubule I and II. Katoh et al. (2008) proposed a regulatory system in the proximal tubules that can switch between secretion and absorption using basolateral and apical NKCC transporters, respectively, depending on their osmoregulatory need in FW or SW. However, the basolateral versus apical locations of NKCC1 and NKCC2 still need to be confirmed in fish tubules.

**FIGURE 3:**
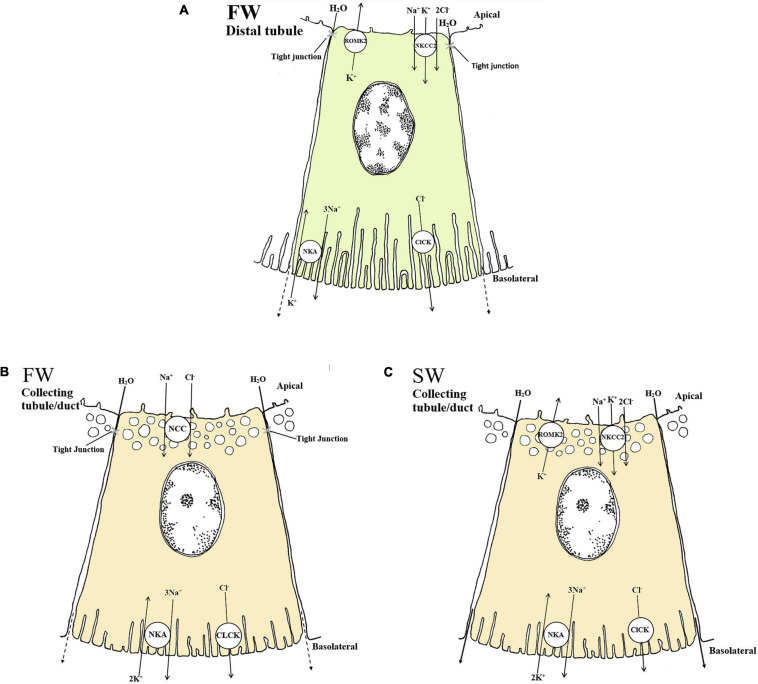
Schematic overview showing localization and mechanisms of Na^+^, Cl^–^ and K^+^ transport in FW **(A and B)** and SW **(C)** teleosts. **(A and B)** The basolateral Na^+^ - K^+^ - ATPase (NKA) generates a favorable transeptial membrane potential used by other ion transporters. In FW urine dilution likely occurs through the apically located NKCC2 in the distal tubule which reabsorbs luminal Na^+^, K^+^ and Cl^–^ through a concentration gradient under water impermeable conditions **(A)**. The cytosolic Cl^–^ are further passed into the interstitial space via the chloride channel (CLCK), while the apical K^+^ channel (ROMK2) maintain the luminal K^+^ concentrations creating a lumen positive transepithelial voltage necessary for Na^+^, K^+^ and Cl^–^ absorption through the NKCC2. In the collecting duct of FW fish NCC further reabsorbs Na^+^ and Cl^–^ aided by the basolateral NKA and CLCK transporters **(B). (C)** In SW the mechanisms of NaCl absorption is likely similar to those in the distal tubule of FW fish, only here the tubules are permeable to water and the NaCl absorption is likely accompanied by water. The ROMK2 and CLCK are based on expression profiles in the pronephric duct of zebra fish (Wingert et al., 2007), tilapia (Miyazaki et al., 2002) kidneys as well as the mammalian model (Nielsen et al., 2002) for all the models. All models are based on the paper of Katoh et al. (2008) and Kato et al. (2011).

The distal tubule function as a Na^+^ and Cl^–^ reabsorbing segment (Hickman and Trump, 1969; Nishimura et al., 1983) where the NKCC2 and ClC-K appear to be critical, thereby contributing to the dilute urine of FW fish (Tipsmark et al., 2002; Wingert et al., 2007; Cutler and Cramb, 2008; Katoh et al., 2008; Kato et al., 2011). The basolateral ClC-K belongs to the ClC family of chloride channels that contribute largely to Na^+^/Cl^–^ reabsorption in the Loop of Henle of the mammalian kidney (Kobayashi et al., 2001; Wojciechowski et al., 2018). Duplicates of the CLC-2 chloride channel are identifed in zebrafish kidney and named *clcn2b* and *clcn2c*, with the latter being substantially higher expressed (Pérez-Rius et al., 2015). A basolateral specific ClC-K in the tubule cells of FW-adapted tilapia indicates a vital role in transepithelial Cl^–^ reabsorption in distal segments (Miyazaki et al., 2002). In zebrafish, a kidney-specific CLC-K channels was located apically in tubule cells, argued to be essential for successful renal reabsorption, (Pérez-Rius et al., 2019), further advocating the importance of CLC-K channels in FW environments in which reabsorption of Cl^–^ are vital. Based on the limited evidence available it appears that both an apical and basolateral specific CLC-K channel are present in the teleost kidney. The CLC-K has also been suggested to operate together with the NKCC2 and NCC in the collecting duct of the euryhaline pufferfish (Kato et al., 2011; Figure 3). *In vitro* studies on FW-acclimated rainbow trout showed that Cl^–^ absorption in the distal tubule is significantly higher than Cl^–^ secretion, suggesting a net Cl^–^ reabsorption in the distal segment (Nishimura et al., 1983). Expression of the *nkcc2*α isoform in the European eel (restricted to the renal tissue, as in mammals) further supports a reabsorptive role in the distal segment in FW (Cutler and Cramb, 2008). Efficient NaCl reabsorption in the distal and collecting segments of FW-acclimated teleosts probably depend on the apical NKCC2 cotransporter and the basolateral NKA pump and ClC-K channels (Miyazaki et al., 2002; Nishimura and Fan, 2003; Cutler and Cramb, 2008; Kato et al., 2011; Pérez-Rius et al., 2019; Figures 3A,B).

### The Solute Carrier 12 Member 3 (SLC12A3, NCC)

The distal tubule and collecting duct of FW euryhaline fishes may be functionally equivalent to the ascending limb and distal convoluted tubule in mammals; the latter reabsorbs Na^+^ through the Epithelial sodium channel, ENaC (Loffing et al., 2001). Surprisingly, the ENaC transporter appears to have been lost in some vertebrate groups such as the ray-finned fishes (Actinoptergii), though lobe finned fishes (Sarcopterygii) have retained the transporter (Hanukoglu and Hanukoglu, 2016), and it has described in the lungfish kidney (Uchiyama et al., 2012). The Solute Carrier 12 member 3 (SLC12A3), also referred to as NCC (sodium chloride cotransporter), is expressed in the collecting tubule/duct of several FW teleosts and is suggested to compensate for the absence of a ENaC (Cutler and Cramb, 2008; Katoh et al., 2008; Kato et al., 2011). While the NKCC2 is thought to reabsorb Na^+^ and Cl^–^, thereby diluting the urine in the distal segment (Kato et al., 2011), the NCC is thought to further reabsorb Na^+^ and Cl^–^ in the collecting tubule/duct and urinary bladder (Gamba et al., 1993; Cutler and Cramb, 2008; Figures 3A,B). However in trout bladder, the use of pharmacological blockers for NKCC (bumetanide) and NCC (chlorothiazide) were ineffective, despite a 50% coupling of Na^+^ and Cl^–^ transport in trout bladder (Burgess et al., 2000) suggesting that more functional studies are required. In FW pufferfish, the NKCC2 was expressed in the distal tubules, while the NCC was expressed in the collecting tubule/duct, likely involved in NaCl reabsorption (Katoh et al., 2008; Kato et al., 2011; Figures 3A,B). During SW acclimation, pufferfish NCC mRNA levels and apical location of the NCC protein were significantly reduced (Kato et al., 2011). The NCC in kidney is essential for the production of hypotonic urine in FW but not in SW (Figure 3B), while the NKCC2 is needed to reabsorb Na^+^ and Cl^–^ in the distal tubule of FW fish and the collecting tubule of SW fish (Katoh et al., 2008; Kato et al., 2011; Figures 3A,C). The closely related NCCα in European eels was downregulated following acclimation to SW (Cutler and Cramb, 2008), which also strengthens the previous conclusion.

### K^+^ Specific Transporters and Channels

Potassium (K^+^) is a major monovalent cation in vertebrates. Basolateral NKA maintains intracellular K^+^ levels roughly 30-fold higher than extracellular K^+^ levels, thereby establishing the membrane potential. There have been extensive reviews on K^+^ handling by the mammalian kidney, involving ROMK, K^+^-ATPase (KA), H^+^-K^+^-ATPase (HKA), Maxi K, KCC1, KCC2 and KCC (Giebisch, 1998; Hebert et al., 2005; Giebisch et al., 2007; Palmer, 2014; Kamel et al., 2018), but information in fish remains sparse. So far, we have focused on NKCC1 and NKCC2 in relation to Na^+^ and Cl^–^ regulation, but K^+^ is also moved by these transporters. Katoh et al. (2008) have proposed that an apically located NKCC absorbs K^+^ in the distal tubule in conjunction with NaCl, aided by ClC-K, K^+^ channels and/or the NKA pump (Figures 3A,C). This is contradictory to the mammalian model where most K^+^ is absorbed in the proximal tubules and the thick ascending limb (Giebisch, 1998; Giebisch et al., 2007). However, NaCl reabsorption in teleosts is suggested to be possible largely due to the lumen positive transepithelial voltages generated by apical renal outer medullary K^+^ channel 2 (ROMK2) (Kato et al., 2011; Figures 3A,C), a hypothesis that needs experimental validation in the teleost kidney. Indeed, parallel to the ROMK2 expressed in the mammalian kidney (Hebert et al., 2005), a ROMK2 ortholog has been identified in the distal segment in zebrafish (*Danio rerio*) larvae (Wingert et al., 2007). Plasma K^+^ levels are very similar in FW and SW teleosts, generally between 2-5 mM (Edwards and Marshall, 2012; Table 1). In the urine the K^+^ levels range from 0.4 to 5.8 mM (Hickman and Trump, 1969; Table 1) and are relatively unaffected by diet compared to other ions (Bucking et al., 2010). While information on renal K^+^ transport in fish remains limited, data on other transport epithelia are emerging. These include expression patterns of ROMK and related pathways such as subfamily M (Maxi-K), K^+^-Cl^–^ cotransporters (KCC1, KCC2) and K^+^-Cl^–^ cotransporter 4 (KCC4) in gills and skin of euryhaline tilapia (Furukawa et al., 2012) and medaka (Horng et al., 2017). Their potential contributions in the kidney warrant investigation.

### Other Important Renal Monovalent Ion Transporters

A portion of Na^+^, Cl^–^ together with glucose are reabsorbed in the proximal segment (Nishimura and Imai, 1982; Dantzler, 2003; Beyenbach, 2004). The pathways remain somewhat unclear but may involve a Na^+^/H^+^ exchanger (Na^+^/H^+^ exchanger 3 (NHE3), SLC9A3) as well as a Na^+^/glucose cotransporter (Solute-carrier family 5 (SLC5A), SGLT) transporter that may facilitate apical entry of Na^+^ into proximal tubule cells (Ivanis et al., 2008). Interestingly NHE3 was localized to the apical membranes of proximal segment II in SW acclimated Japanese eel, and the authors suggested that reabsorption of monovalent ions in the kidney of SW eels is likely mediated by NHE3 while the NKKC2 and NCC (termed NKCC2α and NCCα in Japanese eel) are important in FW eels (Teranishi et al., 2013). However, NHE3 was also detected in FW eel, and thus may operate in FW as well. Nevertheless, most NaCl are reabsorbed in distal and collecting tubule through the NKCC/NCC transporters (previous paragraphs). NHE3 also contributes to renal acid-base regulation in FW rainbow trout, while the SGLT proteins involved in Na^+^/glucose cotransport have not been investigated in teleosts, though are present in cartilaginous fish (Althoff et al., 2006, 2007; Ivanis et al., 2008). In addition, Na^+^ appears to be transported together with phosphate by the type-II sodium-phosphate cotransporter (NaPi-II), now termed SLC34A2B, at the basolateral membrane of proximal segment II, whereas in the collecting tubule and collecting duct, the same transporter is located on the apical membranes and appears to drive reabsorption (Gupta and Renfro, 1989; Elger et al., 1998; Verri and Werner, 2019). Verri and Werner (2019) provide a detailed review of phosphate transport in the kidney. Finally, cystic fibrosis transmembrane conductor (CFTR) has been credited with a secretory role in gill Cl^–^ transport (Cutler and Cramb, 2001; Marshall and Singer, 2002; Hiroi and McCormick, 2012; Wong et al., 2016). Initially *cftr* mRNA was not detected in the eel kidney (Marshall and Singer, 2002), but was later detected in the distal segment of Atlantic salmon (Madsen et al., 2020). Both of the above studies was not able to distinguish between the *cftr1* and *cftr2* isoforms in the kidney. Thus, the role of *cftr* in the kidney, relative to the important contributions of the SLC 12 family and CLC-K transporters (Katoh et al., 2008; Kato et al., 2011), still remains elusive.

The kidney plays an important accessory role to the primary role of the gills in acid-base and ammonia regulation, and monovalent ion transporters are critically involved in these processes in the renal tubules. In addition to NHE3, these include V-type H^+^ATPase, Na^+^-HCO_3_^–^ co-transporter (NBC1), carbonic anhydrase (CA), CAc, CA IV, anion exchanger (AE1), Rhesus (RH) glycoproteins). We consider this to be outside the scope for the current review but is a focus of another detailed review in progress, and will consequently not be addressed here.

### Summary and Knowledge Gaps in Monovalent Ion Transport of the Kidney

The acclimation of euryhaline species to changing salinity must involve a finely controlled regulation of many monovalent transporters. In summary, the NKCC1 seems to be secretory and is largely found in proximal tubule II, while the NKCC2 appears to be reabsorptive in FW and SW, especially in the distal and collecting tubule (Figures 3A,C), with some species-specific variation amongst euryhaline species. Some of these assumptions are based on the use of a general NKCC antibody where the authors refer to a secretory (NKCC1) and reabsorbing (NKCC2) type (Katoh et al., 2008). We assume that the paper of Kato et al. (2011) refers to the collecting tubule when they use the name “early” collecting duct as this are more in line with common anatomical investigations (Hickman and Trump, 1969; Braun and Dantzler, 2011; Dantzler, 2016). The NCC transporter is predominantly found in the collecting tubule/duct and urinary bladder, likely reabsorbing even more Na^+^ and Cl^–^ resulting in a very dilute urine in FW species (Curtis and Wood, 1991; Kato et al., 2011; Figure 3B). Additionally, the teleost NKCC2 and NCC appear to have analogous Na^+^ and Cl^–^ reabsorptive functions to those in the mammalian ascending limb of Henle and distal convoluted tubule, while K^+^ regulation remains less understood. Indeed, much of our understanding of all these transport processes still relies on mammalian models, and only a few euryhaline fish have been studied in detail (eel, tilapia, killifish and pufferfish). Our limited knowledge of NHE3, SGLT and CFTR transporters in the kidney of fish is a particular deficit. More studies on existing species and inclusion of other species are crucial to better understand the kidney’s role in monovalent regulation and its connection to water transport (see next section).

## Aquaporins (AQP) and Their Role in Water Transport in the Kidney of Euryhaline Fish

The aquaporins, first identified in mammals, constitute several intrinsic proteins (MIPs) that facilitate the passive movement of water molecules across cellular membranes (King et al., 2004). AQP functions and regulation are complicated, with many aspects yet to be elucidated (Verkman and Mitra, 2000; Abedin et al., 2019). Vertebrate aquaporins are heterogeneous, found in diverse tissues, and generally categorized based on their permeability preferences for water, glycerol, and other small solutes (Verkman and Mitra, 2000; Takata et al., 2004). Of the 10 AQP isoforms so far described, 7 are present in the mammalian kidney, located along the nephron and collecting duct, emphasizing their importance for renal water handling (Knepper et al., 2001).

Several teleost AQPs has been identified and annotated based on their mammalian analogs: AQP-1aa, -1ab, -3a, -3b, -7, -8aa, -8ab, -9a, -10a, 10b, and -12 (currently known water transporters). Even though several are expressed in the teleost kidney (Tingaud-Sequeira et al., 2010), their roles in renal physiology remain elusive (Cerdà and Finn, 2010). However, recent investigations have shed some light on AQP1, AQP3, AQP8, AQP10, AQP11 and AQP12 regulation and role in euryhaline fish (Martinez et al., 2005; Tipsmark et al., 2010; Engelund and Madsen, 2011, 2015; Madsen et al., 2015; Madsen et al., 2020).

### Aquaporin 1

**FIGURE 4:**
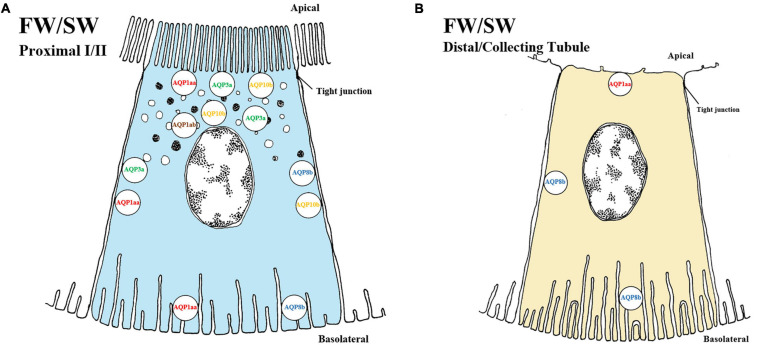
Schematic overview showing localization of AQP (aquaporins) in kidney of teleosts. The localization of several AQP transporters in both proximal **(A)** and distal/collecting **(B)** segments of the kidney. No difference is observed between FW and SW environments of any of the above AQP in the fish kidney. The AQPs are located in apical, sub-apical, lateral and basolateral positions in proximal tubules while no sub-apical location of AQPs has been found in the distal/collecting tubule. The approximate location of each AQP is as follows: AQP1aa (red, apical/lateral/basolateral), AQP1ab (brown, sub-apical), AQP3a (green, apical/sub-apical/lateral), AQP8b (blue, lateral/basolateral) and AQP10b (yellow, apical/sub-apical/lateral). In the distal/collecting tubule only AQP1aa and AQP8b have been discovered/located. AQP distribution in proximal tubule I and II as well as distal and collecting tubule have not been differentiated and need to be experimentally determined. Models are based on the paper of Engelund and Madsen (2015).

Aquaporin 1 proteins participate in water reabsorption from the tubular lumen and may be crucial to avoid dehydration in SW teleosts. In seabass (*Dicentrarchus labrax*) and marine medaka (*Oryzias melastigma*) *aqp1a* mRNA levels are elevated in SW-acclimated fish compared to fish in FW (Giffard-Mena et al., 2007, 2011; Kim et al., 2014). In climbing perch (*Anabas testudineus*) *aqp1a* was induced after 24 h in SW (Ip et al., 2013). Interestingly, reciprocal expression of *aqp1aa* and *aqp1ab* paralogs suggests an even greater regulatory plasticity in Atlantic salmon, with increasing *aqp1aa* mRNA and decreasing *aqp1ab* mRNA levels during smoltification and after SW exposure (Tipsmark et al., 2010), despite conflicting regulation patterns in other species. For instance, in European eel, mRNA levels of both *aqp1aa* and *aqp1ab* paralogs decreased after SW transfer (Martinez et al., 2005). Conversely, mRNA levels of *aqp1* were undetectable in the kidney of SW-adapted Japanese eel (Aoki et al., 2003), while no changes in kidney *aqp1a* mRNA levels were observed in silver seabream (*Sparus sarba)* acclimated from SW (33 ppt) to salinities ranging from 0 ppt to 70 ppt (Deane et al., 2011). In the black porgy (*Acanthopagrus schlegeli)*, renal mRNA levels of *aqp1* were downregulated in FW and upregulated in response to increased salinity (10 ‰) (An et al., 2008). In salmonids, Aqp1aa is located in the proximal tubule and suggested to be a trans-cellular pathway for water movement, while inconsistent expression in the distal tubule supports the notion that the distal segment is impermeable to water (Engelund and Madsen, 2015) (Figures 4A,B). However, AQP1ab is mainly detected intracellularly in sub apical vesicles near the plasma membrane of proximal tubules of FW salmonids (Engelund and Madsen, 2011, 2015) (Figure 4A) and AQP1 was only found apically in undefined tubule cells in European (silver) eels in both FW and SW (Martinez et al., 2005). The proximal tubule location of AQP1 paralogs in salmonids is consistent with that of proximal tubules and the descending limbs of the loop of Henle in mammalian nephrons (Knepper et al., 2001). However, apart from Atlantic salmon, AQP1 has yet to be localized to a specific nephron segment in other euryhaline fish (Cerdà and Finn, 2010).

### Aquaporin 3

In mammals, AQP3 is located on basolateral membranes of tubule cells and seems to have a role in water reabsorption (Knepper et al., 2001). Although mRNA expression of *aqp3* was not initially detected in teleosts (Cutler and Cramb, 2002a), these authors later localized AQP3 in the apical pole of renal tubule cells upon SW-acclimation in eels. There were no differences in protein abundance or location during transition from FW to SW, suggesting a role independent of external salinity (Cutler et al., 2007).

In tilapia and Atlantic salmon renal *aqp3a* mRNA expressions were elevated in SW compared to FW (Watanabe et al., 2005; Tipsmark et al., 2010). However, unchanged kidney *aqp3a* mRNA levels during smoltification supports the notion that regulation of *aqp3a* is not part of the preparatory process prior to SW exposure, but rather responsive to external salinity, as in tilapia (Tipsmark et al., 2010). In Atlantic salmon, AQP3a is expressed in the apical, subapical and lateral space of the proximal tubule and been suggested to have a role in regulation of cell volume through intracellular vesicles and transcellular water transport (Engelund and Madsen, 2015) (Figure 4A). The authors further suggested that the AQP3a might have a role in water conservation or in secondary transcellular water transport. However, in Japanese medaka the mRNA levels of *aqp3a* appeared to be unaffected by salinity (Madsen et al., 2014). Notably, renal *aqp3* (later referred to as aqp3b by Engelund and Madsen, 2011) was not expressed in Japanese eel kidney (Kim et al., 2010) in SW. Hence, it seems that *aqp3b* is either expressed at very low levels, or possibly absent in the fish kidney. In summary, AQP3a channels are probably important for conserving water to avoid dehydration in SW acclimated fish. In contrast AQP3b channels are present in FW European eel but unresponsive to salinity, and expressed only at low levels. Despite the high degree of regulatory plasticity of AQP3a, the exact role of the AQP3b and therefore the AQP3 in general remains uncertain.

### Aquaporin 8

The function of AQP8 in water homeostasis is still elusive, even in mammals where it is predominantly present in liver, intestinal tissue, and intracellular vesicles in proximal tubules and collecting ducts (Elkjær et al., 2001). In salmonids and the Japanese medaka both *aqp8aa* and *aqp8ab* paralogs are present in kidney, although regulatory responses are modest in response to salinity (Tipsmark et al., 2010; Kim et al., 2014; Madsen et al., 2014). The predominant location of AQP8 paralogs (*aqp8aa*, *aqp8ab* and *aqp8b*) is in the liver and intestine of euryhaline teleosts (Engelund et al., 2013). Since none of the paralogs in the kidney of euryhaline species seems particularly responsive to salinity, and protein levels remained unchanged from FW to SW the *aqp8* might be constitutively expressed, rather than associated with hyper- or hypo- osmoregulation (Engelund and Madsen, 2015; Madsen et al., 2015). AQP8b is basolaterally and laterally located in proximal and distal tubules in rainbow trout and Atlantic salmon (Engelund and Madsen, 2011, 2015; Figures 4A,B), possibly serving as a transcellular exit pathway for reabsorbed water to re-enter the extracellular fluids.

### Aquaporin 10

In mammals, AQP10 is not expressed in kidney, but rather located in apical membranes of the small intestine and suggested to participate in the transport of small solutes (Hatakeyama et al., 2001). In the kidney of the stenohaline zebrafish, *aqp10* is expressed as two paralogs, *aqp10a* and *aqp10b* (Cerdà and Finn, 2010). Functional assays using Xenopus oocytes showed that teleost *aqp10* likely is capable of water, glycerol and urea uptake (Santos et al., 2004; MacIver et al., 2009). In euryhaline species only *aqp10b* has been discovered, and reports on mRNA expression are contradictory. Following transfer of Japanese and European eel to SW, *aqp10b* levels were downregulated (MacIver et al., 2009) whilst in Atlantic salmon a fivefold increase was found 8 days post SW transfer (Tipsmark et al., 2010). However, in a later study, Engelund and Madsen (2015) were unable to confirm similar upregulation of *aqp10b* expression following SW transfer. This was consistent with no upregulation of AQP10 at the protein level following SW exposure, suggesting that the AQP10b may have dual regulation, being active independent of external salinity. In eels and Atlantic salmon, the AQP10b channels are located on the apical, sub-apical and lateral membranes of the proximal tubule cells (Figure 4A), suggesting that they facilitate both exit and entry pathways for transcellular water transport (MacIver et al., 2009; Tingaud-Sequeira et al., 2010; Engelund and Madsen, 2015). In summary, while information on the role AQP10 in kidney water transport is limited and unclear, it is likely important for both FW- and SW-acclimated fish.

### Other AQP‘S in Euryhaline Species

Several other aquaporins, including AQP2, Aquaglyceroporins 7 and 9, AQP11a and AQP12, are either present in the teleost kidney but unresponsive to salinity changes, or else described only in mammals (Engelund and Madsen, 2011, 2015).

### Paracellular Transport and Tight-Junction Proteins (Claudins)

Tight junctions proteins (claudins) may supplement AQP-mediated transporters (addressed above) in water transport where they provide a paracellular route. In the gills a large review has been dedicated to the importance of paracellular permeability with respect to environmental factors (Chasiotis et al., 2012). In the kidney of euryhaline teleosts, there is little information on water transport by claudins but CLDN2 have been observed to create water pores in the mouse proximal renal tubules (Rosenthal et al., 2010; Schnermann et al., 2013). CLDN2 has not been investigated in the fish kidney. CLDN15a has been identified in salmon, but its permeability properties remains unknown (Madsen et al., 2020). The authors also discuss the possible involvement of other tight junction claudins (CLDN3, CLDN7, CLDN8, CLDN10, CLDN12, CLDN28, CLDN30) in paracellular transport of both mono and divalent ions (see following section).

### Summary of AQPs and Their Relation to Ion Transport

Engelund and co-authors have contributed extensively to our current understanding of aquaporins in all osmoregulatory organs of euryhaline teleosts, including the possible involvement of other tight junction claudins (CLDN3, CLDN7, CLDN8, CLDN10, CLDN12, CLDN28, and CLDN30) in paracellular transport of both mono and divalent ions (see papers by Engelund and Madsen, 2011; Madsen et al., 2015, 2020). Nevertheless, the current review highlights the lack of knowledge on the distribution and role(s) of AQPs in water and ion transport in the teleost kidney (Figures 4A,B). The close linkages between the transport of Na^+^, Cl^–^ and water in proximal tubules and reabsorption in distal segments are crucial aspects for future studies. The prevailing hypothesis, at least in euryhaline species, is that AQPs likely serve a fundamental role in transcellular water transport in nephron tubules (Madsen et al., 2015). However, very few AQPs have been located in the distal tubules and collecting duct of fish where the primary functions are reabsorption of monovalent ions. Presumably, lack of water channels here would be adaptive for life in FW, allowing for minimal water reabsorption and thus facilitate excretion of excess water. In contrast, permeability to water in these segments would be crucial for water absorption in SW, so as to facilitate a low volume of isotonic urine rich in Mg^2+^, Ca^2+^ and SO_4_^2–^ (Beyenbach, 2004). Functional connections between AQPs, claudins, monovalent ion handling (previous section), and divalent ion handling (see next section) are a rich area for future investigation in the kidney of euryhaline fish (Schnermann et al., 2013; Gong and Hou, 2017; Madsen et al., 2020).

## Divalent Ions and the Known Transporters in the Kidney of Euryhaline Fish

The concentrations of Mg^2+^, SO_4_^2–^, and Ca^2+^ in SW may be up to 100-fold greater than in FW. Vertebrates in general maintain relatively constant concentrations of these divalent ions (1–3 mM) in their extracellular fluids, so SW fish must excrete them by producing urine which contains high concentrations (See Table 1; Smith, 1930; Beyenbach, 2004; Marshall and Grosell, 2005). The kidney is the main excretion pathway, thus euryhaline fish must shift from reabsorption in FW to net secretion in SW, largely via the proximal tubules (Cliff and Beyenbach, 1992; Beyenbach, 1995; Renfro, 1999). To date, only a few euryhaline species are studied, so much of our assumptions are drawn from comparative knowledge from mammalian kidney models. (Markovich, 2001; Dawson et al., 2003; Markovich and Aronson, 2007; Arjona et al., 2019; Chen et al., 2020).

## Sulfate (So_4_^2–^) Transport

Urine concentrations of SO_4_^2–^ have been used as a marker for renal failure in humans, thus the transport pathways have been widely examined in the mammalian kidney (Markovich, 2001; Dawson et al., 2003; Markovich and Aronson, 2007). In FW, SO_4_^2–^ concentrations are generally low (< 1.0 mM) while in SW, SO_4_^2–^ is the second most abundant anion (approx. 26 mM). FW teleosts actively take up SO_4_^2–^ from the environment, whereas in SW, there is an unavoidable influx of SO_4_^2–^ through the gills, and perhaps slight uptake in the gut: 97% of the excess SO_4_^2–^ is excreted through the kidney (Watanabe and Takei, 2012). Plasma SO_4_^2–^ concentrations close to 1 mM must be maintained, regardless of the external environment, so species migrating from FW to SW must make a regulatory shift from renal reabsorption to secretion (Watanabe and Takei, 2011a). The regulation of SO_4_^2–^ has been addressed in flounder, goosefish, eel, pufferfish, rainbow trout and killifish (Hickman, 1968a; Hickman, 1968b; Renfro and Pritchard, 1983; Cliff and Beyenbach, 1992; Renfro et al., 1999; Pelis and Renfro, 2003; Katoh et al., 2006; Kato et al., 2009; Watanabe and Takei, 2011a), with the most extensive investigations in eel and pufferfish (Nakada et al., 2005; Kato et al., 2009; Watanabe and Takei, 2011a, b; Kato and Watanabe, 2016).

### Solute Carrier Family 26 Member 6 (SLC26A6; Apical Transport)

**FIGURE 5:**
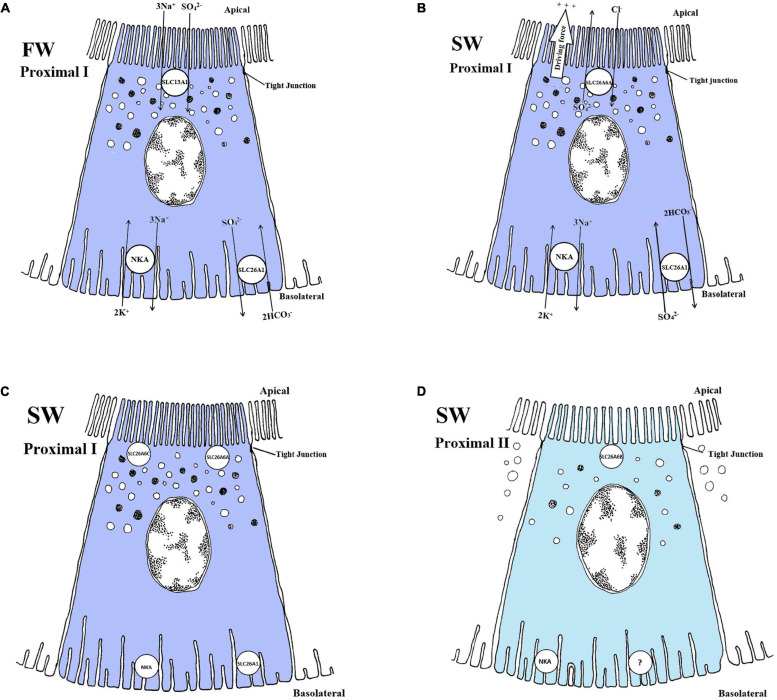
Schematic overview showing localization and mechanism of SO_4_^2–^ transport in FW **(A)** and SW **(B, C and D)** teleosts. **(A)** SLC13A1 is located on apical membranes, co-transporting 3Na^+^/SO_4_^2–^, while SLC26A1 is an electroneutral SO_4_^2–^/HCO_3_^–^ exchanger located on basolateral membranes, driven by Na^+^, K^+^-ATPase (NKA) creating a transmembrane Na^+^ gradient. This transport is located in proximal tubule cells. **(B)** SLC26A6A is an electrogenic SO_4_^2–^/Cl^–^ exchanger located apically, while the SLC26A1 are able to switch the exchange ratio in SW so that it exchanges SO_4_^2–^/HCO_3_^–^ in the opposite direction of FW. NKA creates an electronegative potential in the cell, favoring the transport in SW. **(C and D)** (only showing location) The apical SLC26A6C, SLC26A6A and the basolateral SLC26A1 are located in proximal tubule I (PI), while SLC26A6B are found apical in the proximal tubule II (PII), respectively. The exchange ratios for SLC26A6B and SLC26A6C are not known. Models based on Nakada et al. (2005) (FW), Kato et al. (2009) (SW) and Watanabe and Takei (2011b) (SW).

In the mammalian kidney, the SLC26A6 transporter has been localized to apical membranes of proximal tubules where it is suggested to exchange numerous anions: oxalate/SO_4_^2–,^ Cl^–^/formate, Cl^–^/oxalate, oxalate/formate, oxalate/oxalate, Cl^–^/HCO_3_^–^ and Cl^–^/OH^–^ (Markovich, 2001; Markovich and Aronson, 2007). In teleosts, the SLC26A6A, SLC26A6B and SLC26A6C paralogs have been described in eel and pufferfish, all of which have been localized to the apical membrane of proximal tubule cells (Kato et al., 2009; Watanabe and Takei, 2011b; Figure 5). In the fish kidney, the prevailing hypothesis has largely been the apparent ability of SO_4_^2–^ transport to be driven by a Cl^–^ gradient (Renfro and Pritchard, 1983; Renfro et al., 1999), which does not occur in the mammalian kidney where it is primarily transported using Na^+^ (Na^+^/SO_4_^2–^, cotransport) or exchanged for HCO_3_^–^ (SO_4_^2–^/HCO_3_^–^, exchanger) (Markovich, 2001; Burckhardt and Burckhardt, 2003). This has been further strengthened by electrophysical studies revealing a 50-200 fold higher electrogenic transport of the SLC26A6A in the eel across cell membrane when expressed in Xenopus oocytes (Kato et al., 2009; Watanabe and Takei, 2011b), thereby displaying the highest SO_4_^2–^ transport activity in the SLC26A6 family. Furthermore, apical localization of SLC26A6A to the proximal tubules concurrent with elevated mRNA levels in both SW eel and pufferfish indicates a predominant role of this transporter in SO_4_^2–^ secretion (Kato et al., 2009; Watanabe and Takei, 2011b; Figure 5B). Possible exchange of SO_4_^2–^ against Cl^–^ by this mechanism has been linked to an increase in plasma Cl^–^ levels in SW, presumably coupled with an unknown Cl^–^ sensor in the circulatory system that in turn upregulates *slc26a6a* (Watanabe and Takei, 2011a) which is located apically in the membrane of proximal tubules (Watanabe and Takei, 2011b), thus facilitating a decrease in plasma SO_4_^2–^ concentrations by secretion into the tubular lumen (Watanabe and Takei, 2011a, b). The recent findings in SW eel and pufferfish resonate well with *in vivo* studies on SO_4_^2–^ transport in the kidney, explaining production of an isotonic urine rich in SO_4_^2–^ (Hickman, 1968b; Renfro and Pritchard, 1983; Renfro, 1999). The other two paralogs in teleost fish, the SLC26A6B and SLC26A6C, have been localized to the apical side of the renal proximal tubule but with no apparent differences in mRNA abundance between FW and SW in eel and pufferfish (Kato et al., 2009; Watanabe and Takei, 2011b; Figures 5C,D). Similar to the SLC26A6A, the SLC26A6B also elicited considerable currents for Cl^–^/SO_4_^2–^ transport across the cell membrane of Xenopus oocytes, though no activity was observed in the SLC26A6C.

To date, these paralogs are teleost-specific (Kato et al., 2009; Watanabe and Takei, 2011b) and only one single SLC26A6 has been found in mammals and Chondrichthyes (Markovich and Aronson, 2007; Hasegawa et al., 2016). In teleosts, all three paralogs have been localized apically in renal proximal segments I (SLC26A6A and SLC26A6C) and II (SLC26A6B), respectively (Watanabe and Takei, 2011b) (Figures 5C,D). The expression and presence of SLC26A6B and SLC26A6C in the apical proximal tubule of both FW and SW acclimated fish points to a possible involvement in entry or exit of SO_4_^2–^, depending on the need for absorption or secretion.

### Solute Carrier Family 13 Member 1 (Slc13a1; Apical Transport)

In the mammalian kidney, the SLC13A1 transporter, also referred to as the Na^+^/SO_4_^2–^cotransporter (NaSi-1), is located in the apical membrane of proximal tubules (Lötscher et al., 1996). The exact stoichiometry has not been found, only that it is an electrogenic, pH-insensitive, high affinity Na^+^-dependent SO_4_^2–^ transporter with a substrate preference for both SO_4_^2–^ (K_*m*_ 93 μM) and Na^+^ (K_*m*_ 16–24 mM), and important for the reabsorption of SO_4_^2–^ (Markovich and Aronson, 2007). During FW acclimation in eels, reabsorption of SO_4_^2–^ in the kidney involves both an apical SLC13A1 and basolateral SLC26A1 transporter (Figure 5A; Nakada et al., 2005). A universal role of the SlC13A1 in reabsorbing sulfate in FW teleosts is still under debate as *slc13a1* expression was not detected in FW pufferfish (Kato et al., 2009). The electrophysical properties of SLC13A1 have yet to be determined, but are assumed to be similar to its mammalian counterpart (Busch et al., 1994). In eels, the electrogenic membrane potential established by the NKA pump enables the SLC13A1 cotransporter to reabsorb Na^+^ and SO_4_^2–^ (Figure 5A). Once entering the cell, the SO_4_^2–^ is further exchanged against 2HCO_3_^–^ into the blood using the basolateral SLC26A1 transporter (Nakada et al., 2005; Figure 5A). As pointed out by the authors, this model is partly based on the mammalian counterpart and FW eels are the only euryhaline species where the SLC13A1 has been detected in the kidney (Kato and Watanabe, 2016). Moreover, eels seems to have much higher plasma SO_4_^2–^ concentration in FW (Farrell and Lutz, 1975) compared to other euryhaline species examined (Watanabe and Takei, 2012). The absorptive features of the SLC13A1 transporter in the eel might reflect such elevated plasma SO_4_^2–^ levels, since both chum salmon (*Oncorhynchus keta*) and tilapia have low levels of SO_4_^2–^, and no SLC13A1 transporter has been identified in these species (Watanabe and Takei, 2012). The SLC13A1 likely facilitates elevated plasma SO_4_^2–^ levels, probably particular important for FW eels, because this species lacks active Cl^–^ uptake at the gills (Nakada et al., 2005; Kato and Watanabe, 2016). Nevertheless, how reabsorption of SO_4_^2–^ in the kidney is accomplished in other euryhaline species remains to be determined.

### Solute Carrier Family 26 Member 1 (Slc26a1; Basolateral Transport)

The solute carrier family 26 member 1 (SLC26A1) or sat-1 is a SO_4_^2–^/anion exchanger mediating SO_4_^2–^ transport into extracellular fluid across the basolateral membrane in exchange for HCO_3_^–^ in mammals (Karniski et al., 1998). However, it was later argued that this exchange could move in both directions, mediating cellular exit or entry of SO_4_^2–^ to or from the blood, depending on either apically located SLC13A1 or apically located SLC26A6 (Markovich, 2001). Recent discoveries have revealed a similar mode of transport for the SLC26A1 in the euryhaline teleosts, with the SLC26A1 being the most probable candidate for basolateral transport of SO_4_^2–^ in the kidneys of both FW and SW fish (Nakada et al., 2005; Kato et al., 2009; Watanabe and Takei, 2011a, b; (Figures 5A,B). Reabsorption of SO_4_^2–^ in eels is attributed to the combined actions of the apical SLC13A1 (previous section) and basolateral SLC26A1 (current section) whereas SO_4_^2–^ secretion is attributed to the combined action of the apical SLC26A6A (previous section) and basolateral SLC26A1 (current section). The SLC26A6A further facilitates SO_4_^2–^ excretion from the cell into the lumen (Figure 5B). (Kato et al., 2009; Watanabe and Takei, 2011a, b). The electroneutral SLC26A1 transporter serves a role in excretion of SO_4_^2–^, exchanging 2HCO_3_^–^/SO_4_^2–^ from the cell to the blood in SW (Katoh et al., 2006; Kato et al., 2009; Figure 5B). Similar to the mammalian basolateral SLC26A1 transporter (Markovich, 2001; Markovich and Aronson, 2007), the teleost ortholog SLC26A1 transporter moves 2 HCO_3_^–^ in exchange for reabsorption of 1 SO_4_^2–^ in FW eels (Nakada et al., 2005; Figure 5A). The SLC26A1 seems to play a role in the movement of SO_4_^2–^ in both directions, either exiting or entering the cell at the basolateral side in FW or SW environments, respectively (Nakada et al., 2005; Kato et al., 2009; Watanabe and Takei, 2011a; Kato and Watanabe, 2016; Figures 5A,B).

### Summary and Knowledge Gaps in SO_4_^2–^ Transport

The reabsorption of SO_4_^2–^ in FW fish is facilitated by an apical SLC13A1 and a basolateral SLC26A1 transporter (Nakada et al., 2005; Figure 5A). In SW fish, SLC26A6A are the most probable candidate for apical transporters involved in SO_4_^2–^ secretion (Figure 5B), but SLC26A6B and SLC26A6C may also be involved (Figures 5C,D). The NKA located in the basolateral membrane of proximal tubules (see NKA section) provides the driving force for the electrogenic Cl^–^/SO_4_^2–^ exchanger (SLC26A6A) by increasing intracellular negative membrane potential (Kato et al., 2009). Finally, a basolateral electroneutral SLC26A1 exchanger for the export of SO_4_^2–^ in exchange for HCO_3_^–^ probably occurs in both directions for FW and SW fish (Nakada et al., 2005; Kato et al., 2009; Figures 5A,B). There is a need for further studies on the transporters involved in both SO_4_^2–^ reabsorption in FW and secretion in SW, especially characterization of orthologs. Few species have been investigated concerning SO_4_^2–^ transport and several assumptions are still based on the mammalian model. In addition, transport pathways to reabsorb SO_4_^2–^ in FW are still in dispute and have only been verified in the eel.

## Magnesium (Mg^2+^) Transport

In mammals Mg^2+^ is reabsorbed in the proximal tubule (25%), the thick ascending limb of the loop of Henle (70%) and in the distal tubule (remainder) by transcellular transport via transient receptor potential melastatin type 6 (TRPM6) and Mg^2+^ channels (Voets et al., 2004). As for other divalent ions, Mg^2+^ transporters in the mammalian kidney have received attention due their potential involvement in pathological conditions, though knowledge gaps remain (Arjona et al., 2019; Chen et al., 2020). In teleosts, the current understanding of Mg^2+^ transport is even more limited, with only a few studies addressing the mechanisms involved (Oikari and Rankin, 1985; Chandra et al., 1997; Beyenbach, 2004; Islam et al., 2013, 2014; Kodzhahinchev et al., 2017).

**FIGURE 6:**
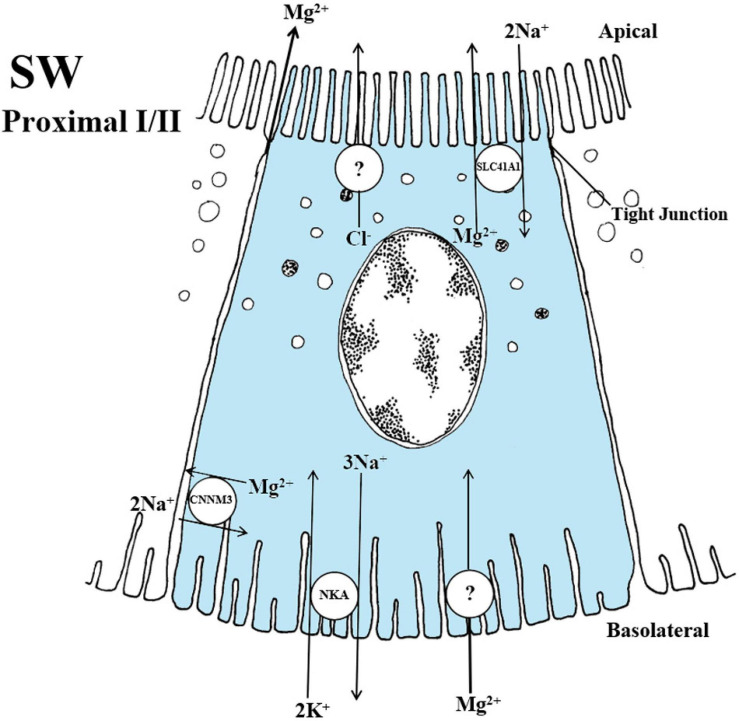
Schematic overview showing localization and mechanisms of Mg^2+^ transport in SW teleosts. The SLC41A1 have been localized to intracellular vesicles that are dispersed apically in the cell of proximal tubule I. In fish, the 2 Na^+^/Mg^2+^ exchange rate has not been verified but has been demonstrated in the human model. The CNNM3 exhibits a lateral localization in both proximal tubules I and II. The specific exchange ratio of 2 Na^+^/Mg^2+^ are again based on the human model whereas the function in fish is still speculative at this point. An apical Cl^–^ channel that produce a lumen-negative transepithelial potential and a basolateral Mg^2+^ channel for transport into the cell are necessary but yet not identified in the fish kidney. The CNNM2 has been linked to Mg^2+^ reabsorption in FW fish however the mechanisms are so premature in the fish model that no overview has been given at this point. Models are based on the papers of Islam et al. (2013), Islam et al. (2014).

Marine teleosts maintain relatively stable plasma Mg^2+^ concentrations at roughly 1.3 mM (Table 1), which require active regulation given the high Mg^2+^ level in SW (∼53 mM) (Smith, 1930; Hickman, 1968b; Hickman and Trump, 1969; Beyenbach, 2004; Marshall and Grosell, 2005). As marine fish excrete even higher Mg^2+^ concentrations (∼57–167 mM) in the urine, this ion needs to be effectively concentrated many-fold from the plasma to urine by the renal system. Secretion of Mg^2+^ by the tubules is thought to occur by basolateral entry along an electrical gradient coupled with apical vesical exocytosis into the lumen (Bijvelds et al., 1997; Renfro, 1999). In addition, a Na^+^/Mg^2+^ exchanger has been hypothesized to aid in Mg^2+^ secretion (Renfro, 1999). To date, two transport families responsible for Mg^2+^ transport have been identified in fish (Figure 6).

### Solute Carrier Family 41 Member 1 (SLC41A1)

The solute carrier family 41 member 1 (SLC41A1) are thought to largely facilitate cellular Mg^2+^ extrusion via Na^+^/Mg^2+^ exchange in mammals, while their potential role in reabsorption remains unclear (Arjona et al., 2018). Xenopus oocytes expressing mammalian SLC41A1 exhibited a Mg^2+^ channel activity (Goytain and Quamme, 2005). Conversely, characterization of human SLC41A1 in HEK293 cells suggests 2Na^+^/Mg^2+^ exchange, representing a different mode of transport of Mg^2+^ (Kolisek et al., 2012; Mastrototaro et al., 2015).

In teleosts, the SLC41A1 transporter has been apically localized to vacuoles in proximal tubules and suggested to be involved in Mg^2+^ secretion (Islam et al., 2013; Kodzhahinchev et al., 2017). In the kidney of the euryhaline pufferfish, *slc41a1* mRNA levels were substantially elevated in SW compared to FW in proximal tubule II, thus indicating a predominant role in SW environments (Islam et al., 2013). The suggested use of vacuolar transport to secrete Mg^2+^ against a tubule concentration gradient would probably involve actin filament assembly modulating vesicular trafficking of Mg^2+^ and exocytosis to the tubule lumen (Hickman and Trump, 1969; Renfro and Shustock, 1985; Chandra et al., 1997; Renfro, 1999; Figure 6). Although Mg^2+^ is normally reabsorbed from the urine in FW fish, in rainbow trout fed a Mg^2+^- enriched diet, there was active secretion (Oikari and Rankin, 1985; Bucking et al., 2010) and increased SLC41A1 mRNA levels in FW goldfish (*Carassius auratus*) exposed to high dietary and environmental Mg^2+^ (Kodzhahinchev et al., 2017). Therefore, it appears that Mg^2+^ can be regulated in relation to changes in both external salinity and diet, likely through the SLC41A1 transporter (Figure 6).

Islam et al. (2013) and Kodzhahinchev et al. (2017) are the first studies to confirm that the SLC41A1 is responsible for vesicular trafficking. Since the SLC41A1 was localized to vacuoles in the apical cytoplasm in proximal tubule II, Mg^2+^ is likely transported from cytosol to the vacuole lumen, and then later exocytosed into the urine (Islam et al., 2013; Figure 6). In contrast, FW rainbow trout brush border membrane vesicles of proximal tubules have been suggested to drive Mg^2+^ reabsorption by an electrical gradient (Freire et al., 1996). The suggestion that SLC41A1 exchanges 2Na^+^/Mg^2+^, in fish, similar to the mammalian counterpart, has yet to be confirmed (Islam et al., 2013).

### The Cyclin M Family (CNNM Family)

The CNNM family also plays a vital role in Mg^2+^ homeostasis in most organisms and is highly conserved evolutionarily (Funato and Miki, 2019). Within the CNNM family, the CNNM1 has been linked to Mg^2+^ efflux, the CNNM4 group has been proposed to be Na^+^/Mg^2+^ exchangers secreting Mg^2+^, while CNNM2 has been linked to reabsorption in mammals.

Several members of the CNNM family appear to undertake specific roles in Mg^2+^ regulation during salinity changes in euryhaline fish. The reciprocal upregulation and downregulation of *cnnm2* transporters in the kidney of FW- and SW-acclimated mefugu (*Takifuga obscurus*), respectively, suggest a role in Mg^2+^ retention in FW (Islam et al., 2014). The stenohaline FW zebrafish fed a Mg^2+^-deficient diet displayed elevated *cnnm2* mRNA levels, which further points to a role in Mg^2+^-reabsorption in FW (Arjona et al., 2013). This is consistent with Mg^2+^ transport in other FW teleosts (Renfro and Shustock, 1985; Bijvelds et al., 1997; Chandra et al., 1997; Beyenbach, 2000) and the reabsorptive function of CNNM2 in mammals (Quamme, 1997; Stuiver et al., 2011; Giménez-Mascarell et al., 2018). Moreover, upregulation of the *cnnm3* transporter in SW-acclimated mefugu kidney points to a possible role in Mg^2+^ secretion across the basolateral membrane of the proximal tubule (Islam et al., 2014). This supports the consensus that CNNM3 has a predominant role in Mg^2+^ secretion in SW-acclimated fish (Beyenbach et al., 1986; Beyenbach, 1995, 2004; Chandra et al., 1997; Figure 6).

### Summary and Knowledge Gaps in Mg^2+^ Transport

In summary, the CNNM2 transporters are currently the most likely candidates for Mg^2+^ reabsorption, while SLC41A1 and CNNM3 are the most likely candidates for secretion, thus combating high Mg^2+^ levels in SW (Figure 6). A potential model for Mg^2+^ transport in FW has yet to be proposed. In mammals, the CNNM family has been linked to Na^+^/Mg^2+^ exchange, though this mode of transport still remains an open question in fish. Hickman and Trump (1969) initially proposed that Mg^2+^ may be linked to water transport by vesicular trafficking in marine teleost nephrons, probably accomplished by an apical located SLC41A1 in vacuoles of proximal tubules (Islam et al., 2013), likely by exocytosis into the tubule lumen. The rapid and marked urinary responses to altered salinity, to Mg^2+^-rich diets and to Mg^2+^ infusions, are all very convincing evidence of euryhaline fishes ability to switch rapidly between absorption and secretion (Oikari and Rankin, 1985; Bucking et al., 2010; Arjona et al., 2013; Kodzhahinchev et al., 2017). Clearly, euryhaline fish are excellent models for the regulation of Mg^2+^ transport. Currently no Mg^2+^ channels have been discovered in teleosts, whereas these serve as key proteins for transcellular transport of Mg^2+^ in mammals, referred to as TRPM6 and TRPM7 channels. Their dysfunction has been linked to hypermagnesemia and secondary hypocalcaemia (Schlingmann et al., 2002, 2007). In mammals, reabsorption of Mg^2+^ has been linked to paracellular transport through tight junction proteins in the claudin protein family 16 and 19 (De Baaij et al., 2012; Claverie-Martin, 2015; Gong and Hou, 2017). Future comparative analysis of teleost claudins (barrier function) CNNM, SLC41, TRPM6 and TRPM7 families in euryhaline species will be informative.

## Calcium

Extracellular fluid Ca^2+^ concentration is tightly regulated in most vertebrates. In terrestrial vertebrates, Ca^2+^ is largely acquired from the diet where whole body Ca^2+^ homeostasis is tightly regulated by the intestinal uptake and renal excretion (Bindels, 1993; Bronner, 2003). As for Mg^2+^, most studies addressing transport mechanisms of Ca^2+^ have been motivated by pathological conditions in humans (On et al., 2008; Gotoh et al., 2015).

Calcium (Ca^2+^) levels in FW range between 0.01–3 mM while SW contains approximately 10 mM (Table 1). In contrast to terrestrial vertebrates, the teleost gills and skin are the main sites of Ca^2+^ uptake; 20-46% originates from gills in FW while the rest is thought to be taken up across the skin (Perry and Wood, 1985; McCormick et al., 1992; Marshall et al., 1995), depending on the developmental stage as larva have higher uptake in the skin when the gills are not fully developed (Hwang, 1996). Fish usually display relatively stable plasma Ca^2+^ levels of approximately 1.5 mM in both FW and SW (Marshall and Grosell, 2005) (Table 1). In FW fish, the kidney generally reabsorbs Ca^2+^, whereas in SW fish, excess Ca^2+^ is secreted, with the urine of SW fish containing relatively high and variable Ca^2+^ concentrations (7.5–39 mM) (Smith, 1930; Wilson et al., 2002; Marshall and Grosell, 2005). However, dietary loading in FW trout resulted in greater Ca^2+^ excretion in the urine due to either less reabsorption from the filtrate, or to increased secretion (Bucking et al., 2010). Several transporters and channels have a potential involvement in Ca^2+^ regulation in the teleost kidney.

### Sodium-Calcium Exchangers (NCX)

The sodium-calcium exchanger (NCX) is a membrane protein within the solute carrier family 8 (SLC8) (Khananshvili, 2013). In humans, the NCX family is involved in cellular Ca^2+^ homeostasis in the intestine, heart, skeletal muscle and nervous system (Loffing et al., 2001; On et al., 2008; Khananshvili, 2013; Liao et al., 2019). Currently, three NCX exchangers have been implicated in Ca^2+^ transport, NCX1, NCX2 and NCX3 (Khananshvili, 2013). Only NCX1 and NCX2 are located in the distal segment of the mammalian kidney and suggested to be involved in Ca^2+^ reabsorption by basolateral electrogenic 3Na^+^/Ca^2+^ exchange in the distal segment (Gotoh et al., 2015; Van Der Hagen et al., 2015; Moor and Bonny, 2016). Although several *ncx* genes have been identified in zebrafish and Pufferfish (Liao et al., 2007; Islam et al., 2011), virtually nothing is known about the regulation and functions of NCX transporters in the teleost kidney.

### Sodium-Calcium Exchanger 1 (NCX1)

**FIGURE 7:**
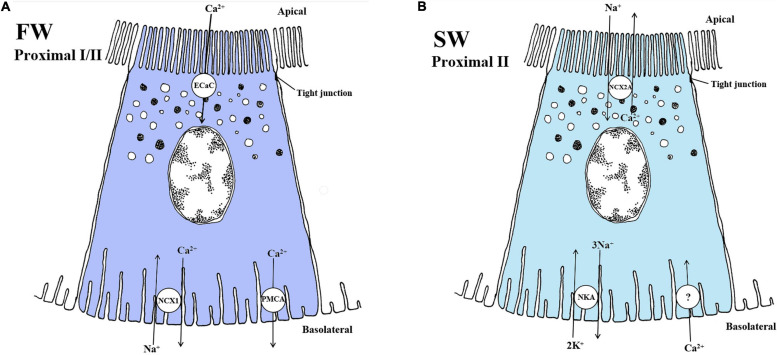
Schematic overview showing localization and mechanisms of Ca^2+^ transport in FW **(A)** and SW **(B)** teleosts. **(A)** In FW fish, the apically located Ca^2+^ channel (ECaC/TRPV5/6) and the basolaterally located NCX1 exchanger Na^+^/Ca^2+^ (NCX1, exchange ratio unknown) likely play a role in reabsorption of Ca^2+^ in the fish kidney. The plasma-membrane Ca^2+^ - ATPase also aids in mediating Ca^2+^ transport from the cytosol to extracellular space, which have been reporter to have high enzyme activity in FW kidney. Thus, models are based on mechanisms in the fish gill and the mammalian kidney model. Understanding of the exact mechanisms is still very limited in the FW teleost kidney. **(B)** In SW fish, the secretion of Ca^2+^ is mediated by the NCX2a in proximal tubule II exchanging Na^+^/Ca^2+^ at the apical membrane. The negative membrane potential and the low intracellular Na^+^ concentration generated by the Na^+^ - K^+^ -ATPase (NKA) are likely the driving forces for this transport. A basolateral Ca^2+^ channel is necessary but has not yet been verified in the fish kidney. Models are based on the papers of Perry et al. (2003); Shahsavarani et al. (2006), Islam et al. (2011), and Sheetal et al. (2018).

In mammals, NCX1 is considered to be essential for basolateral Ca^2+^ transport for both reabsorption and secretion in the mammalian distal convoluted tubule (White et al., 1996; Magyar et al., 2002). In zebrafish, torafugu and mefugu pufferfish, the *ncx1b* is expressed in several organs, including the osmoregulatory gill and kidney (Liao et al., 2007; Islam et al., 2011), while the *ncx1a* is expressed only in the torafugu kidney but not in the mefugu and zebrafish kidney (Islam et al., 2011). Information on NCX1 isoform regulation and function in the teleost kidney is sparce. Nevertheless, based on the mammalian model, one may hypothesize that a basolateral NCX1 is involved in Ca^2+^ reabsorption in FW fish (Figure 7A).

### The Sodium-Calcium Exchanger 2a (NCX2a)

The *ncx2a* is abundantly expressed in the kidney of SW pufferfish, but not in FW zebrafish (Liao et al., 2007). NCX2a is vital for renal Ca^2+^ secretion across the apical membranes into the proximal tubule lumen in conjunction with a Na^+–^dependent exchange, thus playing a significant role in regulating whole body Ca^2+^ homeostasis in SW teleosts (Islam et al., 2011; Figure 7B). Interestingly, thermodynamic calculations suggest that the NCX2a may facilitate reabsorption of Ca^2+^ when primary urine Ca^2+^ concentration is higher than intracellular Ca^2+^ and intracellular Na^+^ is high (Islam et al., 2011), permitting great plasticity in efflux and influx of Ca^2+^ in teleost kidney.

### Plasma Membrane Ca^2+^ ATPase (PMCA) and Epithelial Calcium Channel (ECaC)

In the mammalian distal convoluted tubule, plasma membrane Ca^2+^-ATPase (PMCA) may mediate Ca^2+^ transport, thus playing a role in regulating intracellular Ca^2+^ concentrations (Magyar et al., 2002). The mammalian PMCA consists of four genes, with PMCA1 and PMCA4 being ubiquitously expressed, while PMCA2 and PMCA3 is more tissue-specific (Domi et al., 2007). The pump consists of mainly four domains; the A-domain (or cytoplasmic domain) is important for phosphorylation processes, the P-domain contains the catalytic core of the pump, the N-domain is an important part of the ATP binding site, and finally the calmodulin-binding domain (CaM-BD) is where the inhibitory binding site is found, freeing it from autoinhibition (Gong et al., 2018). The PMCA pump has not been well studied in fish. In the kidney, PCMA isoform regulation could be a important regulatory mechanism of Ca^2+^ transport. However, Ca^2+^ regulation related to dietary Ca^2+^ and environmental levels points to an involvement of both a Na^+^/Ca^2+^ exchanger (NCX; previous section) and the Ca^2+^ ATPase pump in reabsorption in fishes (Verbost et al., 1994; Flik et al., 1996; Figure 7A). Further, a decrease in the Ca^2+^ ATPase enzyme activity in the kidney of tilapia during FW to SW transfer was assumed to reflect a reduced requirement for Ca^2+^ reabsorption. Although plasma membrane Ca^2+^ ATPase (PMCA) activity has been measured in gills, intestine and kidney of teleosts (Sheetal et al., 2018) potential differential regulation of PCMA isoforms remains elusive.

The ECaC is known as a member of the transient receptor potential (TRP) family in which two have been identified (TRPV5 and TRPV6 channels) (Peng et al., 2018). The ECaCs have been suggested to be similar to the TRPV5 and TRPV6 channels found in mammals. In fish gills ECaC appears to play a key role in Ca^2+^ uptake (Perry et al., 2003; Shahsavarani et al., 2006; Figure 7A). In the lake sturgeon (*Acipencer fulvescens*), *ECac* expression was detected in the kidney but at much lower expression levels than in gills. Nevertheless, *ECaC* expression increased in both kidney and gills of sturgeon when exposed to high water calcium conditions while no increase were observed under low calcium levels (Allen et al., 2011). This contradicts upregulation of *ECaC* under low calcium levels in teleosts. One explanation can be that the high Ca^2+^ conditions was grater than *in vivo* circulating Ca^2+^ levels of the lake sturgeon. Apart from the study by Allen and co-authors little is known about the ECaC in the FW fish kidney.

### Summary for Ca^2+^ Transport

While the roles of the gills and intestine in Ca^2+^ transport are relatively well characterized (Marshall, 2002; Wilson et al., 2002; Shahsavarani et al., 2006; Liao et al., 2007; Bucking et al., 2010; Kodzhahinchev et al., 2018), Ca^2+^ transport mechanisms in the teleost kidney remain poorly understood. Nevertheless, the dominant role of NCX1 in Ca^2+^ reabsorption in mammals and the presence of NCX1 in FW zebrafish suggest a similar reabsorptive role in FW- acclimated fish. Ca^2+^, like Mg^2+^, may be transported paracellularly in mammals by claudin proteins involved in barrier function (Moor and Bonny, 2016; Gong and Hou, 2017). In teleosts, however, most studies have focused on the gills. Therefore, future investigations should focus on the kidney, especially for NCX, ECaC, claudins, and Ca^2+^ ATPase (PMCA) (Flik et al., 1996; Pan et al., 2005; Liao et al., 2007) to elucidate how euryhaline teleosts accomplish Ca^2+^ homeostasis in both FW and SW environments.

## Future Research Directions

**FIGURE 8:**
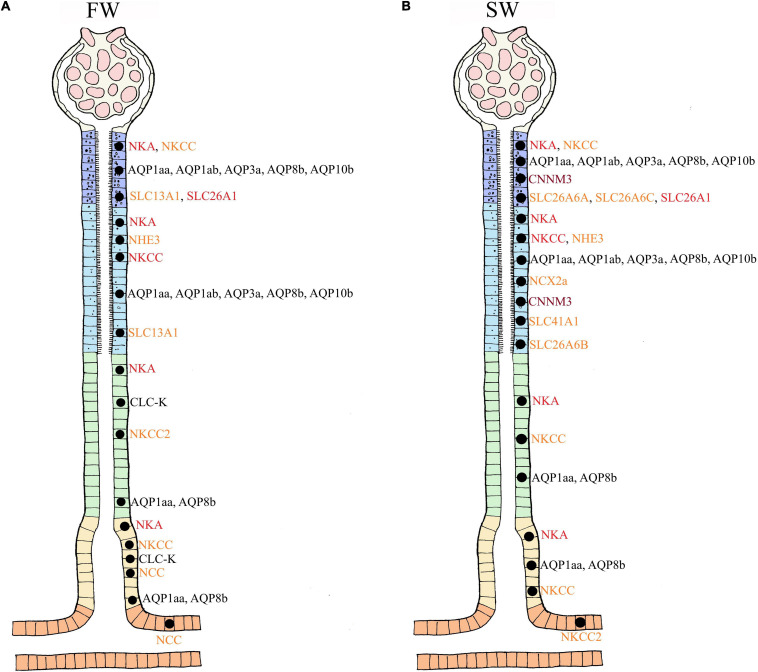
Overview summary of localization of the transporters involved in ion and water transport in euryhaline FW **(A)** and SW **(B)** teleosts. Based on the current molecular analysis in teleosts, locations in the nephron are presented in FW and SW environments. For the following transporters, the experimental analysis has not distinguished between proximal tubule I and II; AQP1aa, AQP1ab, AQP3a, AQP8b, AQP10b, SLC13A1. The NKCC in proximal tubule I/II and distal/collecting duct are based on the detection of a general NKCC antibody which do not distinguish between NKCC1 and NKCC2. The NKCC2 was later detected only in the distal tubule of FW fish and not in the proximal tubule I or II. The NKCC1 may therefore be present in proximal tubules, but specific antibodies still need to verify this. The remaining transporters have been located specifically to the segments presented here in FW **(A)** and SW **(B)** conditions. So far, only NCC (FW) and NKCC2 (SW) have been located in the collecting duct, but likely have a reabsorbtive function (Na^+^, Cl^–^, water) similar to that observed in the collecting tubule. Apical (orange), basolateral (dark red), and multiple locations (black).

Many aspects of tubular transport in the kidney of euryhaline species are complex and remain elusive at the gene and molecular level. Further, the integration of known aspects of renal physiology at the macro level with emerging opportunities provided by modern genetic and molecular methods could further illuminate the role and regulation of the many transporter pathways located in the fish kidney. The localization of all known transporters in teleosts is outlined in both FW (Figure 8A) and SW (Figure 8B).

### Expression Profiling Along the Nephron

Section-specific profiling of gene and protein expression along the length of the fish nephron where sections are separated by either manual (Fehsenfeld and Wood, 2018) or laser capture micro-dissection (Madsen et al., 2020) is promising in providing detailed location and expression profiles of relevant transporters involved in solute and water transport in proximal *versus* distal tubules. Combined with the application of scanning ion-selective electrode technique (SIET) and experimental treatments (e.g., Fehsenfeld et al., 2019), these approaches should be pursued with a range of euryhaline species and treatments.

### Integrative Studies on the Kidney‘s Functional Role in Osmoregulation

Unfortunately, recent advances in immunochemical and molecular approaches have been accompanied by a decline in the application of whole organism physiological approaches to understanding renal function in fish. Only by simultaneously collecting urine, measuring its flow rate (UFR) and composition, as well as the glomerular filtration rate (GFR) by which it was formed, can the experimenter determine rates of secretion and reabsorption of different solutes (e.g., Lawrence et al., 2015). This becomes a powerful approach when combined with section-specific transporter mRNA and protein localization by means of *in situ* hybridization and immunocytochemistry, both of which are useful in determining cellular localization. Several transporters for ion and water regulation addressed in the current review are probably located and function in the same cell(s). Colocalization of transporters will be important for understanding the cooperative effort of different transporters in individual cells throughout the nephron. Future studies should also take a more holistic view, including the integrated osmoregulatory roles of the gill, intestine and kidney as they work in conjunction to maintain overall homeostasis. For example, an overwhelming fraction of the studies on renal function in fish have been performed on fasted animals, whereas we now know that nutrients and ions in the diet have marked effects on transport functions in the intestine, gills, and kidney (Wood and Bucking, 2011).

### Comparative Studies on Mammals and Teleosts

New findings on transporters in the mammalian kidney should be pursued to find homologies in the fish kidney. Mammalian research is more extensive and better funded due to the requirement for understanding the many underlying pathological conditions of renal physiology in humans. Benefits may also flow in the other direction: the plasticity of euryhaline fish, and their ability to adapt to very different ionic environments may provide powerful experimental models to explain regulation and abnormalities of ion and water transport in the mammalian kidney. This may also help understanding how stenohaline fish, that regulate in a much narrower salinity range will cope with changes in salinity related global climate change. Furthermore, euryhaline species that have undergone additional whole genome duplications (WGD) provide intriguing models to study the regulation of paralog genes and their corresponding proteins (Bailey et al., 1978; Houston and Macqueen, 2019).

### Applications for Aquaculture (Diseases, Water Quality, and Diet)

Traditionally, some of the most devastating diseases attacking euryhaline species in aquaculture have been kidney diseases – for example proliferative kidney disease (PKD; Hedrick et al., 1993), bacterial kidney disease (BKD; Fryer and Sanders, 1981), nephrocalcinosis (Fivelstad et al., 2018), and haemorrhagic smolt syndrome (HSS; Byrne et al., 1998). The implementation of effective recirculating aquaculture systems (RAS) and increasing intensification in modern aquaculture is arguably an important driver in the increasing prevalence of adverse effects on fish (Tang et al., 2009; Dalsgaard et al., 2013; Skov, 2019). Such adverse effects are even more prevalent and challenging when farming species, for instance salmonids, that require specific environmental conditions for development (photoperiod, temperature, salinity) to set their developmental trajectories during their lifecycle (Stefansson et al., 1993; Duncan et al., 1999; Fjelldal et al., 2011; Imsland et al., 2014; Strand et al., 2018). Further, innovations in commercial feed, including supplementation with a variety of electrolytes will increase pressures on renal function. Indeed, dietary Na^+^, Cl^–^, Ca^2+^, Mg^2+,^ and phosphate all have potential implications for growth, health status, acid-base balance, and osmoregulation in fish (Salman and Eddy, 1988; Sugiura and Ferraris, 2004; Bucking and Wood, 2007; Ferreira and Baldisserotto, 2007; Cooper and Wilson, 2008; Bucking et al., 2010; Wood and Bucking, 2011; Bucking et al., 2013; Kodzhahinchev et al., 2017). Thus, increasing our mechanistic knowledge of the kidney‘s role in osmoregulation from organismal through molecular levels will be critical in addressing these issues, and thereby protecting and promoting aquacultural productivity of euryhaline species such as eel, tilapia and salmonids.

## Author Contributions

MT and TN drafted the first version of the manuscript. CW actively contributed to writing of the manuscript. HK made the anatomical drawings of the kidney. All authors contributed to the article and approved the submitted version.

## Conflict of Interest

The authors declare that the research was conducted in the absence of any commercial or financial relationships that could be construed as a potential conflict of interest.
